# Genipin-Based Crosslinking of Jellyfish Collagen 3D Hydrogels

**DOI:** 10.3390/gels7040238

**Published:** 2021-11-27

**Authors:** Laura Riacci, Angela Sorriento, Leonardo Ricotti

**Affiliations:** 1 The BioRobotics Institute, Scuola Superiore Sant’Anna, 56127 Pisa, Italy; leonardo.ricotti@santannapisa.it; 2 Department of Excellence in Robotics & AI, Scuola Superiore Sant’Anna, 56127 Pisa, Italy

**Keywords:** jellyfish collagen, hydrogels, crosslinking, cell-laden biomaterials, chondrocytes

## Abstract

Collagen-based hydrogels are an attractive option in the field of cartilage regeneration with features of high biocompatibility and low immunogenic response. Crosslinking treatments are often employed to create stable 3D gels that can support and facilitate cell embodiment. In this study, we explored the properties of JellaGel™, a novel jellyfish material extracted from *Rhizostoma pulmo*. In particular, we analyzed the influence of genipin, a natural crosslinker, on the formation of 3D stable JellaGel™ hydrogels embedding human chondrocytes. Three concentrations of genipin were used for this purpose (1 mM, 2.5 mM, and 5 mM). Morphological, thermal, and mechanical properties were investigated for the crosslinked materials. The metabolic activity of embedded chondrocytes was also evaluated at different time points (3, 7, and 14 days). Non-crosslinked hydrogels resulted in an unstable matrix, while genipin-crosslinked hydrogels resulted in a stable matrix, without significant changes in their properties; their collagen network revealed characteristic dimensions in the order of 20 µm, while their denaturation temperature was 57 °C. After 7 and 14 days of culture, chondrocytes showed a significantly higher metabolic activity within the hydrogels crosslinked with 1 mM genipin, compared to those crosslinked with 5 mM genipin.

## 1. Introduction

Collagen is one of the main components of the connective tissue extracellular matrix; it is a natural substrate for the support and growth of a variety of cells and tissues in the body and acts as a structure in conjunction with other extracellular molecules, such as glycosaminoglycans and fibronectin. Due to its properties (excellent biocompatibility, low antigenicity, and biodegradability) it has been broadly explored in the field of tissue engineering [1,2]. Collagen has been classified into two main categories, based on their supramolecular structures: non-fibrillar collagens and fibrillar collagens. The non-fibrillar collagens belong to a family of structurally related short-chain proteins that do not form large fibril bundles. Instead, the fibrillar collagens form highly organized fibers and fibrils, providing structural support for the body in the skeleton, skin, hollow organs, and capsules from different organs. Fibrils are mainly organized into bundles or lamellae; the size and higher-order arrangement of fibrils give rise to tissue-specific biomechanical and other biological properties. The fibrillar collagens include type I, II, III, V, and XI. Type I collagen is produced by fibroblasts and other cells, such as osteoblasts; as a key component of the extracellular matrix, it is abundant in bone, tendon, skin, ligaments, and cornea, and comprises between 80% and 99% of the total body collagen. Type II collagen is the major collagen type in cartilaginous tissues, although it is also present in other connective tissues, such as the *nucleus pulposus* of the intervertebral disk and the vitreous humor. Type III collagen is a normal constituent of the skin (10–20% of the total collagen) and it is found in many other connective tissues; it provides resistance to forces and stretching. Type V collagen is abundant in vascular tissues [3], while type XI collagen is found in cartilaginous tissue [3].

Collagen as a biomaterial is typically extracted from mammalians (especially from dermis, tendons, and bones that are rich in fibrillar collagen). Although purified collagen can be isolated from human peripheral tissues or human placenta, animal species, such as rats, bovines, pigs, and sheep are often the preferred source [4]. In particular, primary sources of animal-derived collagen are bovine skin and tendons and porcine skin. However, animal-derived collagen presents many disadvantages related to possible immunogenicity and transmission of diseases, such as bovine spongiform encephalopathy [5]. Moreover, some patients refuse to receive components derived from those animals, for ethical and religious reasons [6].

Some marine organisms (e.g., the jellyfish) have a high content of fibrillar collagen (more than 60% of their weight), representing an intriguing alternative for collagen extraction. Jellyfish-derived collagen shows high biocompatibility and low immunogenic response [7]. These properties, together with ease of handling [6] and large bioavailability, make jellyfish collagen an excellent candidate for replacing mammalian one [8]. Jellyfish collagen also shows promising features for cartilage regeneration; indeed, it has been proven that chondrocytes preserve their phenotype when included in three-dimensional (3D) matrices made of jellyfish collagen [6,9]. Collagen from different jellyfish species shows similarities to the collagen of different vertebrates. In fact, it has been shown that some jellyfish collagens are comparable to vertebrate collagen IV or V, while others resemble vertebrate collagen I [10]. Collagen derived from *Rhizostoma pulmo* has shown a high degree of similarity with mammalian type I collagen, but also showing collagen type II- “like” properties.

In this paper, we focused on JellaGel™, a new type of jellyfish collagen, extracted from *Rhizostoma pulmo* and belonging to the category of fibrillar collagen. JellaGel™ was recently marketed and specifically formulated to form three-dimensional gels, thus seeming interesting for tissue engineering applications. Since JellaGel™ is a new and rather unexplored material, almost no works on it are available in the literature, except from a preliminary study reporting the expression of cellular filopodia within the hydrogel matrix, although it was not crosslinked [11].

Crosslinking treatments are frequently used to create stable 3D scaffolds (including collagen ones) supporting cell encapsulation. Indeed, crosslinking is an important aspect that would turn otherwise weak gels into more robust materials, enhancing their stability over time. Genipin is a hydrolytic product of geniposide isolated from the fruits of *Gardenia jasminoides* and it is classified as a natural crosslinking agent [12]. It can spontaneously react with the amine groups of amino acids (including those constituting collagen) to form dark blue pigments [13]. The reaction between genipin and collagen induces the formation of cyclic structures, which enable intramolecular and intermolecular crosslinks [14]. The cytotoxicity of genipin is considerably lower than that of other chemical crosslinking reagents, such as glutaraldehyde (that is 5000–10,000 times more cytotoxic). Accordingly, the biocompatibility of materials crosslinked through genipin is superior to those crosslinked by glutaraldehyde or epoxy compounds [13]. In the literature, genipin-crosslinked collagen matrices (of animal origin) have shown good viability of chondrocytes or stem cells seeded on them [12,15].

To the best of our knowledge, no works concerning genipin-based crosslinking of JellaGel™ are available in the literature. The aim of this paper is to investigate the influence of genipin in the formation of 3D crosslinked JellaGel™ hydrogels and on the embodiment of human chondrocytes. Three genipin concentrations were tested and for each concentration material properties were assessed. In particular, Scanning Electron Microscope (SEM) and Differential Scanning Calorimetry (DSC) were used to analyze the morphological and thermal properties of the hydrogels, respectively. Rheometric measurements were used to characterize the matrix mechanical properties. Finally, the influence of different genipin concentrations on the metabolic activity of chondrocytes embedded within the JellaGel™ hydrogels was assessed at different time points (3, 7, and 14 days).

## 2. Materials and Methods

### 2.1. Hydrogel Preparation

JellaGel™ solution was purchased by the company Jellagen Marine Biotechnologies (Cardiff, UK) and prepared according to the manufacturer’s instructions, as reported in the following. A solution of 10× Phosphate Buffered Saline (PBS, P4417, Sigma–Aldrich, Darmstadt, Germany) was first prepared dissolving one tablet in 20 mL of deionized water. Then, 25 mg/L of Phenol red (P3532, Sigma–Aldrich) were added. For each sample, 222 µL of such solution were mixed to 2 mL of JellaGel™ solution in a glass vial, thus reaching a ratio of 9:1 between JellaGel™ and PBS. The pH of the solution was adjusted until the pH range was 7.5–8.5 using sodium hydroxide solution (S5881, Sigma–Aldrich) at 2M and 0.2M.

To improve the stability of collagen hydrogels, genipin was used as a crosslinker at different concentrations. First, 125 mg of genipin powder (G4796, Sigma–Aldrich) were dissolved in 12.5 mL of Dulbecco’s Phosphate Buffered Saline modified without calcium and magnesium chloride (DPBS, D8537, Sigma–Aldrich) to achieve a final genipin concentration of 10 mg/mL. After adjusting the pH of the JellaGel™ solution as described above, the genipin solution was added in an appropriate quantity to reach the final concentrations of 1 mM, 2.5 mM, and 5 mM, under constant stirring for 3 min. The solution was left at room temperature for 15 min and then incubated at 37 °C overnight to allow gel formation. After the overnight incubation, the samples (without cells embedded) were used for SEM, DSC, and rheometric analyses.

**Figure 1:**
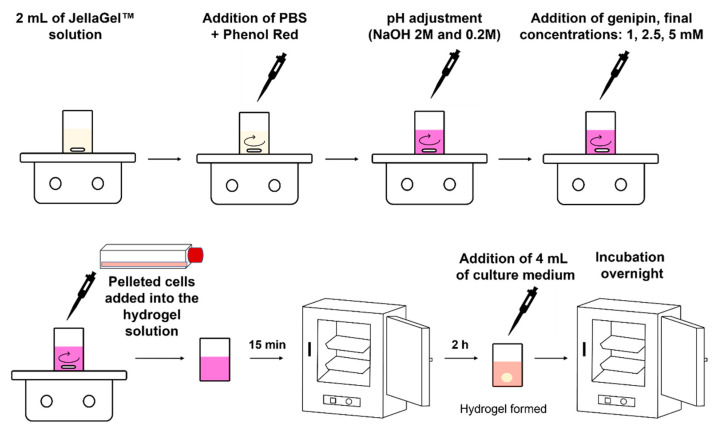
Graphical representation of the hydrogel preparation procedure: the different phases needed for preparing the hydrogels starting from JellaGel™ solution and the key parameters for each step are depicted.

To investigate cell metabolic activity, chondrocytes were encapsulated in the 3D crosslinked JellaGel™ hydrogels, as follows. Human Chondrocytes (HC), derived from normal human articular cartilage, were purchased from Cell Applications (Cat. number 402-05a) and expanded in a human chondrocyte Growth Medium (Cell Applications, San Diego, California, Cat. number 411-500) at 37 °C and 5% CO_2_ atmosphere. To obtain cell-laden hydrogels, the pelleted cells were first resuspended in 50 µL of medium and then added in 2 mL of JellaGel™ solution with a final density of 400,000 cells/mL. Before starting the hydrogel preparation procedure described above, all the solutions were filtered using a filter with a nominal pore diameter of 0.22 µm (16532-K, Sartorius) and the vials and the stir bars were sterilized in the autoclave for 90 min at a temperature of 121 °C and a pressure of 1 atm. The cells were evenly mixed in the solution through a magnetic stirrer at a velocity of 400 rpm. After 3 min, the stirring was stopped and the solution containing the cells was left at room temperature for 15 min. Then, the compound was gently transferred (without shaking it) into an incubator (37 °C and 5% CO_2_). After 2 h, each sample was provided with 4 mL of cell medium and then kept in the incubator overnight. The samples were entirely immersed in the medium but the bottom side detached from the culture well, allowing the scaffold to float just below the medium level. This guaranteed a supply of oxygen and nutrients on all hydrogel sides. The medium was changed every two days. The sample preparation procedure is depicted in Figure 1.

### 2.2. SEM Imaging

SEM imaging was used to analyze the morphology of one representative sample for each genipin concentration. After the preparation described above, the sample (without cells) was dried overnight and gold-sputtered before SEM acquisition. SEM scans were carried out using an EVO MA10 SEM microscope (Zeiss), setting a beam voltage of 5 kV, a current of 90 pA at a working distance of around 10 mm. The images were obtained using a magnification of 1600x. A morphometric analysis was performed on SEM images to evaluate the diameter of the formed collagen fibers. For the measurement of the fiber diameters, we used the open source tool DiameterJ (https://imagej.net/plugins/diameterj, accessed on 10 November 2021), already validated for this purpose [16]. DiameterJ follows a two-step process: (i) first, several image segmentations in a binary image are provided starting from the original image; (ii) then, the analysis of the segmented images is performed. For each image, four segmented images were analyzed, and the final histogram was derived from the sum of frequencies of the four segmented images. A weighted mean and weighted standard deviation were also calculated for each experimental condition.

### 2.3. DSC Analysis

DSC was used to investigate the effect of chemical crosslinking on the thermal characteristics of jellyfish collagen. Three samples (without cells) for each genipin concentration were used for this analysis. The samples were freeze-dried and analyzed through a DSC system 1 STAR (Mettler Toledo), with a heating rate of 2 °C min^−1^ and a temperature range from 25 °C to 75 °C. An empty melting pan was used as the reference sample.

### 2.4. Rheometric Measurements

Rheometric analyses were performed on three independent samples for each genipin concentration, using a rheometer (Anton Paar MCR-302) at a temperature of 25 °C in a plate–plate geometry, with a diameter of 25 mm and a gap between the two plates of 1 mm. After the preparation, the samples (without cells) were gently transferred on the rheometer plate to start the measurements.

The storage modulus (G’), the loss modulus (G”) and the shear stress (τ) were measured in oscillation mode from 0.01 to 1000% strain at a frequency of 1 Hz and the results were plotted in a log-linear scale graph by using GraphPad 8.0.2.

### 2.5. Assessment of Chondrocyte Metabolic Activity

Cell metabolic activity was assessed after 3, 7, and 14 days on JellaGel™ samples with embedded chondrocytes, by using the PrestoBlue™ Cell Viability Reagent (A13261, Invitrogen). Three concentrations of genipin (1, 2.5 and 5 mM) and three samples for each concentration were tested to investigate the effect of the crosslinking level on metabolic activity. The same samples were analyzed at different time points (3, 7, and 14 days). Before each metabolic analysis, the samples were moved to a 6-well plate to avoid any possible contribution to the metabolic activity outcome due to cells not embedded in the hydrogel, but rather attached at the bottom of the vial or in the supernatant. PrestoBlue™ is a resazurin-based solution that uses the reducing ability of live cells to quantitatively evaluate metabolic activity. When cells are alive and healthy, they maintain a reduced environment within their cytosol. Upon entering a living cell, the PrestoBlue™ reagent is reduced to resorufin, which is red in color and highly fluorescent. Metabolically active cells continuously convert the PrestoBlue™ reagent and they can be monitored by measuring the change in fluorescence. Non metabolically active cells cannot reduce the dye and thus they do not generate a change in the signal [17].

At the desired time-points, PrestoBlue™ was first diluted with culture medium in a ratio 1:10. Then, 2 mL of such solution were added to each sample. After incubation for 2 h at 37 °C, the solution was split into 4 wells (300 µL for each sample), obtaining a total of 12 measurements (three independent samples and four measurements for each sample) for each genipin concentration and each time-point. A VICTOR Multilabel plate reader (PerkinElmer, Waltham, MA, USA) was used to read the fluorescence signal, setting an excitation wavelength of 560 nm and an emission wavelength of 590 nm.

### 2.6. Statistical Analyses

For DSC, rheometric and metabolic tests, three independent samples for each genipin concentration were prepared and characterized. A Kolmogorov–Smirnov test allowed assessing the non-normality of data distribution. The statistical comparison between the samples crosslinked with different genipin concentrations, at each time point, was performed using a non-parametric Kruskal–Wallis test, whereas a post-hoc test was performed using a non-parametric Mann–Whitney test for unpaired data. Statistical significance was corrected for multiple comparisons according to the Bonferroni–Holm rule and a *p*-value of 0.05 was set as the significance threshold.

## 3. Results

### 3.1. Preparation of JellaGel^TM^ Hydrogels

**Figure 2:**
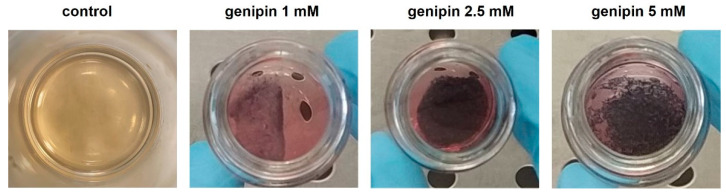
JellaGel™ hydrogels crosslinked with genipin at different concentrations. One representative sample for the control (no genipin) and for each genipin concentration is shown. The dark structures visible in the images correspond to the collagen gel networks, formed due to the bonds established between the collagen chains.

Figure 2 shows representative images for the control (JellaGel™ without the addition of genipin) and samples crosslinked with different genipin concentrations. The samples featured by higher concentrations of genipin (2.5 mM and 5 mM) showed better-formed collagen networks than the ones obtained with a genipin concentration of 1 mM. Samples not provided with genipin (control) were not able to form a *stable* gel. For such a reason, we excluded control samples from the subsequent analyses.

**Figure 3:**
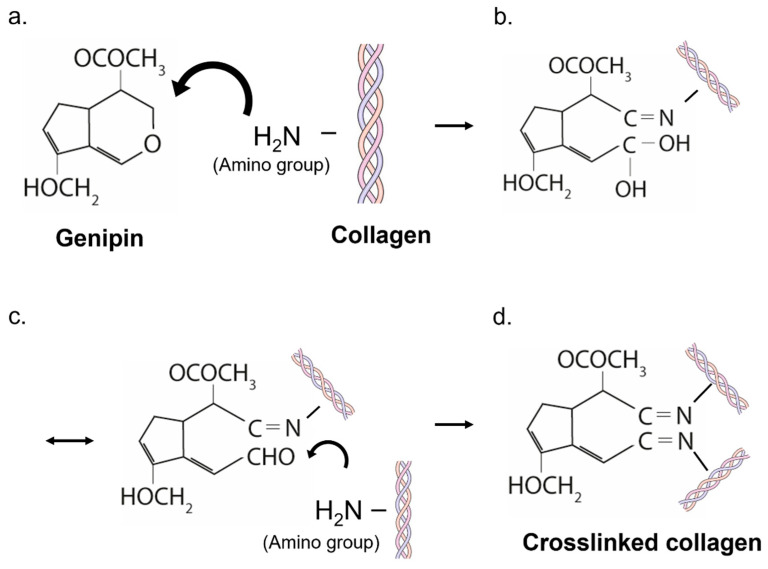
Schematic representation of the crosslinking mechanism of genipin. (**a**) genipin interaction with primary amine groups; (**b**) ring-opening reaction in genipin and covalent bond with the amino group of collagen; (**c**) formation of an unstable intermediate aldehyde group; (**d**) formation of a new covalent bond with another polimer, which leads to the formation of the crosslink.

A schematic representation of the crosslinking mechanism between genipin and collagen is depicted in Figure 3. The extraction of genipin and its chemical reaction with natural biomaterials (such as collagen) has been extensively studied in the past, thanks to the promising biosafety and specific crosslinking performances of genipin [18]. Genipin is able to crosslink materials that contain primary amine groups (Figure 3a). Although genipin is colorless, the release of dark blue pigments occurs when it reacts with the primary amines. The crosslinking mechanism starts with a ring-opening reaction caused by the amino group via a nucleophilic attack on the olefinic carbon atom of the genipin (Figure 3b). Consequently, the genipin forms a covalent bond with the amino group of the polymer (Figure 3b). An unstable intermediate aldehyde group is formed, and it is again attacked by another amine group from another polymer (Figure 3c), forming a new covalent bond, which leads to the formation of the crosslink (Figure 3d) [19].

### 3.2. SEM Imaging

**Figure 4:**
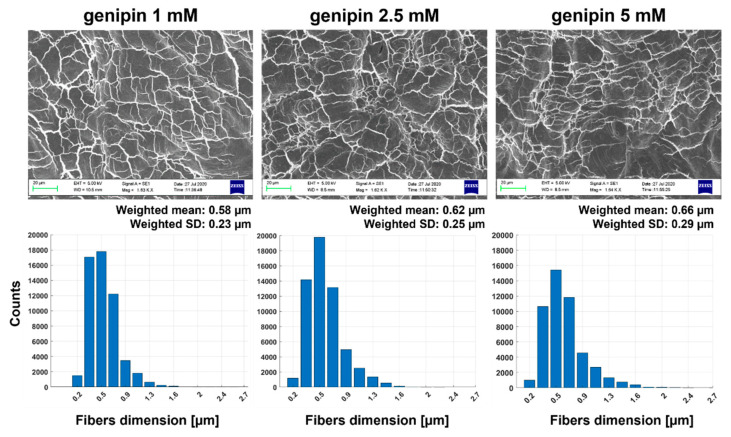
SEM images of JellaGel™ hydrogels crosslinked with genipin at different concentrations. The images are reported at a magnification of 1600×. They show the internal gel microarchitecture with the domains created by the collagen network. For each concentration, a histogram showing the diameter of the collagen fibers is also reported, highlighting the weighted mean and standard deviation for each concentration. Scale bars = 20 µm.

In Figure 4, representative SEM images are reported for the different sample types crosslinked with genipin.

The diameters of the collagen fibers formed in 3D JellaGel™ hydrogels resulted in the order of hundreds of nanometers. In particular, the weighted mean values of the fiber diameters were 0.58 µm, 0.62 µm, and 0.66 µm for 1 mM, 2.5 mM and 5 mM genipin concentrations, respectively. No considerable differences in the collagen fiber microstructure were observed for the different genipin concentrations; the dimensions and organization of the network of fibers were similar for all the experimental conditions, as confirmed by the corresponding histograms.

### 3.3. DSC Analysis

**Figure 5:**
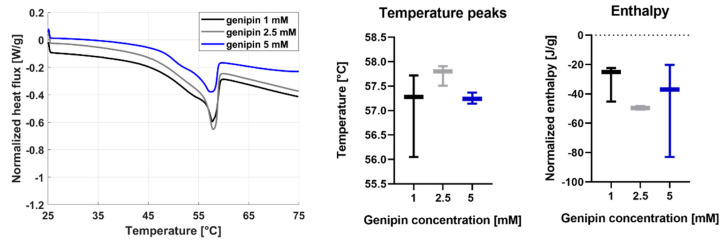
Results of the DSC analysis on the JellaGel™ hydrogels crosslinked with genipin at different concentrations. Heat flux vs. temperature curves are shown for one representative sample of each genipin concentration. The temperature peak and enthalpy are also reported for all sample types.

The DSC analysis was performed on three dried samples for each genipin concentration. The results are shown in Figure 5.

The mean temperature peak was similar for all the genipin concentrations: 57.0 °C for 1 mM, 57.7 °C for 2.5 mM, and 57.3 °C for 5 mM. No significant differences in the temperature peak values were found, thus indicating no influence of the crosslinking degree on the denaturation temperature. The enthalpy change was also calculated by integrating the area under the thermogram, to determine the energy of the bonds keeping the protein in the folded conformation. Also, enthalpy values did not show significant differences for the different genipin concentrations. A relatively high variability of the enthalpy values was observed, especially for the 5 mM genipin concentration (−47.7 ± 32 J/g).

### 3.4. Rheometric Measurements

**Figure 6:**
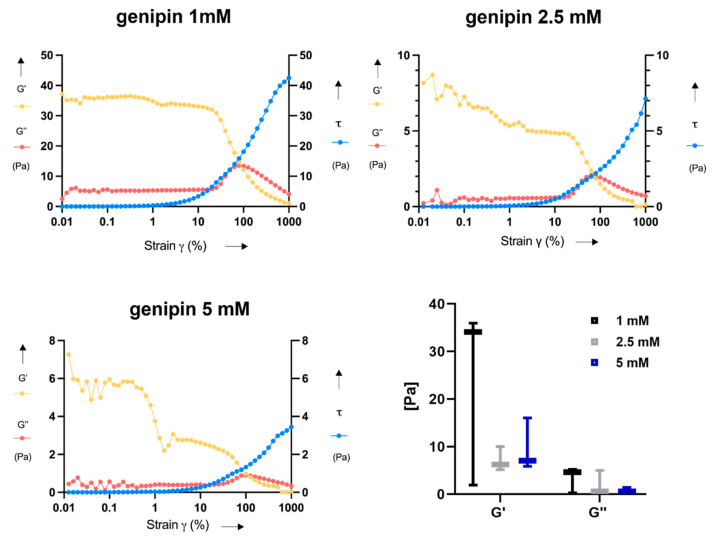
Results of rheometric measurements. Representative graphs of the trend of storage modulus (G’), loss modulus (G”), and shear stress (τ) versus the shear strain (γ) are reported for each genipin concentration. Boxplots with G’ and G” values, calculated in the linear region of the curves, are also reported for all samples at the three genipin concentrations.

In Figure 6, the results of rheometric measurements in terms of storage modulus (G’), loss modulus (G”), and shear stress (τ) are shown. From the representative curves shown for each sample type, it can be observed that the crossing point between G’ and G” occurs at ~100% strain. More in detail, G’ was higher than G” at low strains, thus indicating a more elastic behavior at strain values smaller than 100%, for all samples. On the other hand, G’ was lower than G” at high strains, turning into a more viscous behavior at strains over 100%. The mean values of G’ and G” were comparable for all the genipin concentrations; indeed, no significant differences were found among the three tested concentrations for G’ and G”. However, the G’ and G” values resulted rather variable among independent samples crosslinked with the same genipin concentration, especially in case of G’ for 1 mM (23.97 ± 19 Pa).

### 3.5. Cell Metabolic Activity

**Figure 7:**
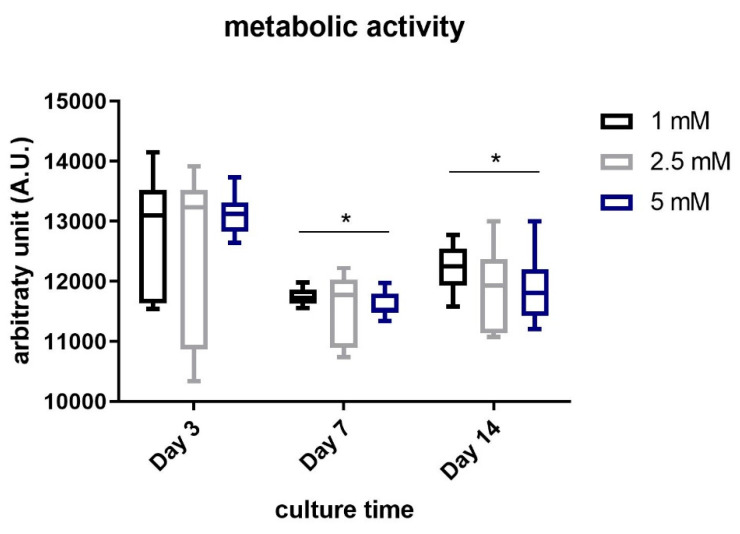
Metabolic activity of human chondrocytes embedded in JellaGel™ hydrogels crosslinked with genipin at different concentrations, after 3, 7, and 14 days of culture. * = *p* < 0.05.

Figure 7 shows the results obtained in terms of metabolic activity of chondrocytes embedded within the JellaGel™ hydrogels crosslinked with the three genipin concentrations, for different culture time-points (3, 7, and 14 days).

After 3 days, no significant differences were found among the three concentrations. The metabolic activity of the cells embedded within the hydrogels crosslinked with 1 mM genipin was significantly (even if slightly) higher than the one observed in the hydrogels crosslinked using 5 mM genipin both at day 7 (*p* = 0.02) and day 14 (*p* = 0.04).

## 4. Discussion

In this work, the influence of three different genipin concentrations (1, 2.5, and 5 mM) on the formation of 3D crosslinked JellaGel™ hydrogels was explored for the first time. One representative gel for each genipin concentration is shown in Figure 2. As mentioned in the Results section, the gels prepared with higher concentrations of genipin (2.5 mM and 5 mM) appeared thicker and better formed than those with a genipin concentration of 1 mM. Genipin results were crucial for obtaining stable gels, which were instead impossible to achieve without any crosslinking agent. This made it impossible to include a non-crosslinked control in the subsequent characterization tests.

The SEM images shown in Figure 3. demonstrated that the different genipin concentrations did not affect the microstructure of collagen fibers within the hydrogels. This finding is in agreement with the state-of-the-art: it has been demonstrated in fact that a crosslinking procedure using different ratios of EDC/NHS did not significantly influence the microstructure of jellyfish collagen samples [20]. Moreover, Figure 3 suggested that the characteristic dimensions of the collagen network structure formed in the JellaGel™ hydrogels were in the order of ~0.6 µm. A direct comparison with previous works is difficult to perform. Indeed, the different collagen sources and the different crosslinking agent used can influence the dimension of the fibers. Moreover, previous similar studies did not perform a quantitative analysis of the diameters of the collagen fibers [21,22]. However, collagen fibers generally present a cord or tape shape 1–20 µm wide. These fibers consist of closely packed thin collagen fibrils (30–100 nm thick), which are organized to form a three-dimensional network as a whole [23]. In our case, we found fiber diameters of hundreds of nanometers, in agreement with the general fibers’ diameters reported in previous literature [23,24]. The results of the DSC analysis are reported in Figure 4. They suggest that the denaturation temperature was not influenced by the crosslinking degree. The temperature peak was found at around 57 °C for all concentrations. The denaturation temperature of pure collagen is about 37 °C [25]. Hence, the crosslinking process, for all genipin concentrations, guaranteed achieving a higher denaturation temperature, allowing gel stability at body temperature. Our findings are in agreement with results reported in the literature. Hoyer et al. [10] found an increase of about 12 K in the denaturation temperature of jellyfish collagen crosslinked with 1% EDC with respect to pure jellyfish collagen. The enthalpy results suggest that the energy needed to keep the proteins in their original state did not depend on the genipin concentration used to crosslink the gel. It is worth mentioning that the enthalpy change depends on the percentage of native proteins in the original solution. Hence, the great variability observed in the enthalpy values may be associated with the variability in the gel formation.

In rheological analyses, the correct loading of the testing plate is an important aspect, which influences the rheological results [26]. Probably, the procedure for sample preparation required for this test negatively influenced the results, increasing their variability; indeed, it was difficult to properly place the samples on the rheometer testing plate without almost destroying them. This aspect can explain the relatively high variability found in the rheological results, presented in Figure 5. Besides rheometric analyses, the encountered manipulability issues are an important aspect in view of possible future pre-clinical and clinical translation of this material.

Finally, we evaluated the metabolic activity of chondrocytes embedded in JellaGel™ hydrogels, as shown in Figure 6. This test could assess the suitability of the JellaGel™ matrix as a possible 3D environment hosting chondrocytes. Results revealed that the cell metabolic activity did not decrease over the culture time, until 14 days. This suggests that the crosslinked matrices can safely host chondrocytes for several days, without hampering nutrient diffusion, not interfering with cell metabolic processes. Eun Song et al. [20] demonstrated that the viability of human chondrocytes seeded on jellyfish collagen scaffolds crosslinked with EDC was stable over time. In our work, we evaluated for the first time the metabolic activity of chondrocytes embedded in JellaGel™ hydrogels crosslinked with different genipin concentrations. Wang et al. [27] investigated cytotoxic effects on chondrocytes incubated with a culture medium containing different genipin concentrations. After 24 h of incubation, the death of chondrocytes exposed to 5 mM genipin was ~ 65% higher than the control. Indeed, 5 mM was identified as the minimum concentration of genipin that can induce toxicity when placed in direct contact with cells. Zhou et al. [28] also demonstrated that the cytotoxicity on adipose stem cells (ASCs) embedded in genipin-crosslinked hydrogels was affected by genipin concentration. In their case, cytotoxicity and proliferation assays demonstrated that the best genipin concentration was 0.02% *w/v* (0.2 mg/mL). In addition, cell proliferation decreased on day 7 and day 14. These results are in agreement with those found in our work: the cells embedded in hydrogels crosslinked with 1 mM genipin (0.226 mg/mL) showed a slight better metabolic activity compared to the other genipin concentrations (especially compared to 5 mM) at 7 (*p* = 0.02) and 14 days (*p* = 0.04).

In previous reports, the potential of jellyfish collagen has been demonstrated in the field of cartilage regeneration [6,29]. JellaGel™ was formulated to create 3D hydrogels providing and maintaining a realistic, near-native microenvironment for cells [11]. However, natural materials are often affected by some limitations related to their usability and reproducibility. JellaGel™ being a natural material, it revealed a rather poor reproducibility in the material preparation procedure. This issue typically affects natural materials that, since they derive from animal sources, often imply a high batch-to-batch variability and also challenging processability [30]. We demonstrated that genipin-based crosslinking allows to obtain stable gels able to embed cells, although with a relatively high variability of the hydrogel properties.

Overall, our results assessed a good biological activity, confirming JellaGel™ as a biocompatible matrix for chondrocytes. On the other hand, results revealed some limitations of this material concerning poor reproducibility in the material processing, high variability, and manipulability issues. These issues should be solved to enable future in vivo applications of this material.

In this work, the authors mainly focused on the physical characterization of crosslinked JellaGel™ hydrogels without chondrocytes embedded, and investigated the influence of the genipin crosslinker on the basic properties of JellaGel™. However, the authors are aware that the inclusion of cells in a 3D hydrogel may alter its properties. Hence, future tests will be needed to perform a more refined characterization of JellaGel™ with embedded chondrocytes, to also evaluate how the cell production of extracellular matrix and the formation of new cartilaginous tissue over time may affect the physical properties of the material.

## 5. Conclusions

In this study, three different genipin concentrations (1 mM, 2.5 mM, and 5 mM) were used to obtain 3D crosslinked JellaGel™ hydrogels. Scanning electron microscopy, differential scanning calorimetry, and rheometry allowed for the analysis of the morphological, thermal, and mechanical properties of the materials, respectively. The hydrogels treated with the highest concentrations (2.5 mM and 5 mM) of genipin appeared by visual inspection thicker and more well-formed than the one crosslinked with 1 mM genipin. Morphological results revealed no changes in the microstructure of the hydrogels increasing the level of genipin concentration. The average diameters of the collagen fibers were 0.58 µm, 0.62 µm, and 0.66 µm for 1 mM, 2.5 mM, and 5 mM genipin concentrations, respectively. Similarly, the denaturation temperature and the enthalpy change of the hydrogels were not affected by the degree of crosslinking. The denaturation temperature result of 57 °C for all the genipin concentrations made genipin-crosslinked JellaGel™ hydrogels stable at body temperature. Rheometric analyses showed no significant differences in the mechanical properties of gels crosslinked with different genipin concentrations, with a crossing point between G’ and G” occurring at ~100% strain. Finally, genipin-crosslinked JellaGel™ hydrogels proved to constitute a suitable matrix for human chondrocyte embodiment. The metabolic activity of the embedded cells did not decrease from day 3 until day 14 of culture. The cells encapsulated in samples crosslinked with 1 mM genipin showed a slightly higher metabolic activity than the others. The material analyzed in this study also revealed some negative aspects, such as a relatively high variability (typical of natural materials), low reproducibility, and low manipulability. New material processing strategies or different crosslinking methods should be devised in the future, to overcome the mentioned limitations.

## Data Availability

The datasets generated during and/or analyzed during the current study are available from the corresponding author on reasonable request.
